# Exploring the Usage Intentions of Wearable Medical Devices: A Demonstration Study

**DOI:** 10.2196/19776

**Published:** 2020-09-18

**Authors:** Chiao-Chen Chang

**Affiliations:** 1 Executive Master Program of Business Administration in Biotechnology College of Management Taipei Medical University Taipei City Taiwan

**Keywords:** wearable medical device, unified theory of acceptance and use of technology, usage intention, health consciousness, trust

## Abstract

**Background:**

In the face of an aging society, an immediate and preventive medical system urgently needs to be established, and the application of wearable devices is essential. However, the application of smart medical care in Taiwan is still not widespread, and few studies have explored the related issues of wearable medical device usage. Thus, determining the success of a wearable medical device mainly depends on the degree of user adoption and use.

**Objective:**

The purpose of this study was to examine the factors that influence the intention to use wearable medical devices.

**Methods:**

This study applied the unified theory of acceptance and use of technology (UTAUT) to build a comprehensive model that explains intentions to use wearable medical devices.

**Results:**

The research findings showed that health consciousness and trust were the strongest predictors of intentions to use wearable medical devices.

**Conclusions:**

The results reveal the magnitudes of the impacts of the variables in a well-accepted revised UTAUT model in the context of the medical industry, particularly in the setting of wearable medical devices. Several important implications for academics and industry decision-makers can be formulated from these results.

## Introduction

### Background

With the emergence of various wearable devices in recent years, the concept and statement of “smart medical care” are gradually emerging in medical innovation. The development of smart medical care has a long history. In addition, with the advancement and rapid rise of the internet of things (IoT) technology, a large amount of medical information has been exchanged and analyzed, which has become the basis of medical big data. Artificial intelligence, which has developed rapidly in recent years, has been introduced as an inductive use of these data. After the combination of the IoT and artificial intelligence, instant mobile medical care emerged, which is the core concept of smart medical care.

Wearable devices can detect the physical condition, use real-time perception, and compare and analyze a large amount of data for analysis, interpretation, and response and can then select the most appropriate current processing and support. Through smart medical care, many dilemmas faced by the current medical system have been resolved. The global market for wearable medical devices is expected to increase from US $6.22 billion in 2017 to nearly US$ 14.41 billion in 2022 at a compound annual growth rate of 18.3% (2017-2022) [1]. Furthermore, the emerging market demand introduced by smart health care is also a big business opportunity.

Further, according to Gartner’s latest forecast [2], by 2020, global end user spending on wearable devices will reach US $51.545 billion, up 27% from US $40.581 billion in 2019. Among them, consumers will spend the most on smartwatches and smart clothing, growing 34% and 52%, respectively. In the past few years, the improvement of sensor accuracy, the development of miniaturization, and better user data protection have made more consumers willing to buy wearable devices. As for hardware manufacturers, they are focusing on sensors that are smaller and smarter, so that the sensors built into wearable devices can obtain more accurate readings, and more usage examples continue to appear. Previous literature has focused on a single form of smart medical service, such as discussing the application of wearable medical services from the perspective of developers [3]. There are few studies considering wearable medical devices from the perspective of users. To fill the abovementioned research gaps, this study developed and validated empirically a model that explicates users’ intentions to use wearable medical devices. Specifically, it revisits a popular contemporary adoption theory (unified theory of acceptance and use of technology [UTAUT] [4]) by augmenting it to better capture wearable medical device environments. Recently, Zhou [5] added a health consciousness construct to the UTAUT in a wearable medical device context. Zahir and Gharleghi [6] also effectively introduced an innovation-related construct (trust), which influences users to adopt the technology. Thus, these two constructs are especially relevant when identifying users’ characteristics regarding the adoption of information technology (IT).

Hence, this study augments the application of the UTAUT and adds two individual factors (health consciousness and trust) to explain users’ intentions regarding wearable medical devices. The purpose of this study was to combine the UTAUT and the two specific factors to improve the IT adoption model and explain the users’ intentions for wearable medical devices.

### Literature Review

#### Wearable Medical Devices

In the face of an aging society, an immediate and preventive medical system urgently needs to be established, and the application of wearable devices is essential. Wearable devices can help patients to detect more serious medical conditions early and then provide early assistance and warning to patients with diseases such as diabetes. This provides an opportunity for people to analyze solutions in health care.

Wearable medical devices include a cardiac sensing electrode, a behavior electrode, a user interface, and a sensor. Indeed, wearable medical devices are designed to diagnose, prevent, and avoid diseases. According to the Food and Drug Administration (FDA), a medical device should not achieve its purposes through chemical action within or on the body, and an agent achieving its purpose through chemical action is termed as a drug.

#### Conceptual Model

##### Revisiting the Main UTAUT

The UTAUT [4] is a technology acceptance model that aims to provide a rough framework specifically designed to explain technology acceptance and use. In particular, this theoretical framework introduces the following two main aspects regarding its predecessor: (1) redefining the four explanatory variables included in the original UTAUT of performance expectancy (PE), effort expectancy (EE), social influence (SI), and facilitating conditions (FC) to adapt them to the consumption context; and (2) identifying three additional key constructs from prior research on both general adoption and use of technologies and consumer adoption and use of technologies. The main constructs in the UTAUT are as follows: PE, EE, SI, and FC. Despite its recent adoption in the literature, the UTAUT has already been tested in some studies that have confirmed its validity to explain technology adoption in consumption contexts, including the wearable medical device industry [7].

##### Intention to Use

Intention to use refers to “the degree to which a person has formulated conscious plans to perform or not perform some specified future behavior” [8]. Furthermore, Venkatesh et al [4] indicated that intention to use is the main indicator of the effectiveness of an information system. The usage intention of wearable medical devices is also a form of information system adoption.

##### Performance Expectancy

PE refers to an individual’s perception that information service (IS) facilitates the completion of a task [4], that is, it means the degree to which users perceive that using wearable medical devices will enable them to achieve improved health management. PE is of direct relevance to the use of wearable devices for medical management in life. This is because users rely on the use of wearable devices to access adequate information. As a result, this study assumed the following hypothesis: hypothesis 1 (H1), PE has a positive influence on the intention to use wearable medical devices.

##### Effort Expectancy

EE is defined as an individual’s evaluation of the effort necessary to complete a task using a given IS [4]. Venkatesh et al [4] viewed EE as the degree of ease associated with the use of an information system. EE is also based on the idea that there are relationships among the effort put forth at work, the performance achieved from that effort, and the rewards received from the effort [9]. Thus, this study proposed the following hypothesis: hypothesis 2 (H2), EE has a positive influence on the intention to use wearable medical devices.

##### Social Influence

SI refers to how an individual perceives the degree of approval of a certain behavior from important referents [4,10]. In addition, SI has a strong origin in attitudinal-behavioral theories (eg, Theory of Reasoned Action [11]), although it was not present in the preceding theories of IS adoption, such as the technology acceptance model (TAM) [12]. Taylor and Todd [13] indicated that peer influence from friends and classmates and superiors’ influence from professors indirectly influenced behavioral intention through the mediator of subjecting norms. Using the medical wearable device would be affected by influences from superiors or important people; therefore, the following hypothesis was proposed: hypothesis 3 (H3), SI has a positive influence on the intention to use wearable medical devices.

##### Facilitating Conditions

FC refers to the degree to which an individual believes that a technical infrastructure exists to support technology use [14]. In commercial settings, FC represents the extent to which a consumer believes that resources exist, and they facilitate the task completion while adopting IS [13]. This construct was introduced more recently in the IS adoption literature to overcome the narrower focus of previous research almost exclusively on a user’s internal belief system [4]. Hence, the following hypothesis was proposed: hypothesis 4 (H4), FC has a positive influence on the intention to use wearable medical devices.

##### Health Consciousness

Health consciousness refers to the degree to which health concerns are integrated into a person’s daily activities and health conscious people are aware of and concerned about their wellness, resulting in better motivation to improve or maintain their health [15]. That is, health consciousness is the degree to which health concerns are integrated into a person’s daily actions [16]. Health conscious people are aware of and concerned about their wellness; therefore, they are motivated to improve and/or maintain their health. The following hypothesis was proposed: hypothesis 5 (H5), health consciousness has a positive influence on the intention to use wearable medical devices.

##### Trust

Trust refers to the belief that someone or something is honest, reliable, good, and operative or the wish to depend on someone or something for security. It represents the intention of a party to be vulnerable to the actions of other parties [17]. Trust becomes a critical issue for research because it plays a role in building satisfied and expected outcomes as a result of a transaction [18]. In the context of mobility, trust has played an important role in explicating the adoption of mobile payment [19]. Similar to other online contexts, trust is a relevant determinant of adoption in the wearable medical device scenario owing to the impersonal nature of the mobile internet environment and the uncertainties involved in such transactions. In line with this assumption, this study proposed the following hypothesis: hypothesis 6 (H6), trust has a positive influence on the intention to use wearable medical devices.

## Methods

### Research Model

**Figure 1:**
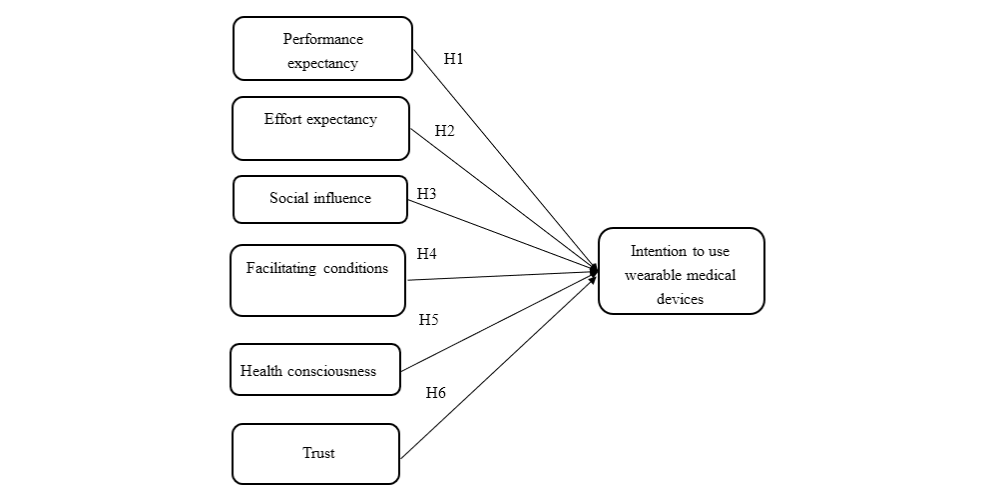
Research model.

The conceptual model for the study was developed from the researcher’s view of the interactions that could exist between the variables of the study based on a review of the literature. The model proposes a direct relationship between the independent variables and the dependent variable. Specifically, it is assumed that there is a relationship between PE and the use of wearable medical devices. In addition, there could be a link between EE and the use of wearable medical devices. It is also evident from the model that a relationship could be proposed between FC and the use of wearable medical devices. In addition, the model also seeks to test the influence of the three independent variables on the dependent variable (Figure 1).

### Instrument Development

For data collection, this study developed a self-administered online survey. Measurement scales for all construct items were taken from existing scales based on prior work [4,20], with modified wordings to adapt the items to the topic area. In the pretest phase, the questionnaire was reviewed by a small group of IS faculty and management students. The scales were modified as a result of their suggestions. The questionnaire was then tested with a sample of medical and business school students and personnel. This resulted in further modifications to the questions. The purposes of these pretests were to confirm that relevant aspects were included and to enhance the clarity and readability of the questionnaire.

### Measurements

To ensure the content validity of the scales used, the items selected should represent the concept around which generalizations are to be made. Items selected for the constructs were therefore largely adapted from prior studies [4] to ensure content validity. In this study, the constructs of the UTAUT were taken from the study by Venkatesh et al [4] and modified to reflect the utility of wearable medical devices, whereas the constructs of health consciousness and trust were taken from the studies by Ahadzadeh et al [15] and Safa and Solms [21].

The participants were instructed to rate each item of the dependent variables on a 5-point Likert scale from 1 (strongly disagree) to 5 (strongly agree).

### Procedures and Participants

This study posted an electronic survey through an online survey platform (Survey Cake) to obtain a sample from the mass population of wearable medical device users. The wearable medical devices included devices and technologies (eg, wearable glucose monitoring and drug delivery devices, activity monitors, smart clothing, smart equipment, wearable vital sign monitors, and smartwatches). Using Facebook, a popular social networking site, potential participants were chosen to complete surveys. After clicking on the link and entering the questionnaire website, participants were considered wearable medical device users or potential users.

### Data Collection and Samples

An online self-administered survey questionnaire was considered an appropriate instrument to identify wearable medical device users. All questions in the questionnaire were measured on a 5-point Likert scale, ranging from “1” (strongly disagree) to “5” (strongly agree). The time required to complete the questionnaire was almost 3 to 5 minutes. The final questionnaire of items is presented in Multimedia Appendix 1 [4,20,21].

All subjects participated in the study voluntarily during the period from July 20 to August 20, 2019. There were 452 participants overall (252 male and 200 female participants). The mean age was 47.8 years, and participants aged 39 to 55 years accounted for 53.1% (240/452) of the study sample. Most of the participants (344/452, 76.1%) stated that they were familiar with the term “wearable medical devices” prior to completing the survey.

### Data Analysis

Structural equations among latent constructs were examined to test the conceptual structural equation model (SEM). The SEM was used to analyze causal models and simultaneously estimate a series of interrelated dependence relationships. Thus, data analysis was carried out using structural estimation modeling. Before this study tested the research model, SPSS 25.0 for Windows (IBM Corp) was used to show the important descriptive information on demographic variables, including participant characteristics such as gender, age, and educational background. This information also included behaviors related to the use of wearable medical devices, such as the time spent on the internet, the preferred online medical platform provider, and the frequency of using wearable medical devices. Model evaluation involved a two-step analysis [22] using the software IBM Amos 21.0. For this purpose, the author first built a measurement model using confirmatory factor analysis for the model to check its fit and then built the SEM and examined the hypothesized causal paths among the constructs by performing a simultaneous test. This helped to observe whether the conceptual framework had provided an acceptable fit to the empirical data.

### Measurement Model

The validity of the measurement model was evaluated by investigating convergent validity, discriminant validity, and reliability. Structural equation modeling has been used to evaluate the plan’s research model and hypotheses. Simultaneously, for assessing the reliability of measurement items, this research computed composite construct reliability coefficients. Therefore, all the average variances extracted exceeded 0.50, all composite reliabilities were larger than 0.70, the factor loadings of all items exceeded the recommended level of 0.60, and all values were significant at .001, demonstrating that the scales had good convergent validity. In addition, the Cronbach α of the seven constructs ranged from .81 to .89. All composite reliabilities were larger than 0.70, displaying good reliability [23]. The results conﬁrmed good reliability (Table 1).

Discriminant validity is shown when (1) measurement items load more strongly on their assigned construct than on the other constructs in a confirmatory factor analysis and (2) the square root of the average variance extracted of a construct is larger than its correlations with the other constructs [24]. To test the discriminant validity, this research computed the square root of the average variance extracted and factor correlation coefficients. For each factor, the square root of the average variance extracted should be greater than its correlation coefficients with other factors to show that the scale has a worthy discriminant validity [25]. As shown in Table 2, all constructs had an average variance extracted value higher than the threshold of 0.50, confirming the convergent validity of the constructs.

**Table 1:** Loading and composite reliability values for the items.

Item			Loading		Composite reliability
**Performance expectancy (PE)**					0.81
	PE1	0.80			
	PE2	0.82			
	PE3	0.81			
	PE4	0.84			
**Effort expectancy (EE)**					0.84
	EE1	0.79			
	EE2	0.86			
	EE3	0.84			
	EE4	0.81			
**Social influence (SI)**					0.86
	SI1	0.83			
	SI2	0.89			
	SI3	0.89			
**Facilitating conditions (FC)**					0.83
	FC1	0.82			
	FC2	0.80			
	FC3	0.85			
**Health consciousness (HC)**					0.89
	HC1	0.90			
	HC2	0.89			
**Trust (TR)**					0.87
	TR1	0.86			
	TR2	0.88			
	TR3	0.88			
**Intention to use (INT)**					0.86
	INT1	0.87			
	INT2	0.80			
	INT3	0.81			
	INT4	0.86			

**Table 2:** Correlations between constructs.

Variable^a^	PE^b^	EE^c^	SI^d^	FC^e^	HC^f^	TR^g^	INT^h^
PE	0.73	—^i^	—	—	—	—	—
EE	0.59	0.79	—	—	—	—	—
SI	0.62	0.63	0.84	—	—	—	—
FC	0.58	0.71	0.82	0.82	—	—	—
HC	0.71	0.70	0.77	0.68	0.83	—	—
TR	0.61	0.60	0.71	0.80	0.77	0.84	—
INT	0.58	0.59	0.58	0.75	0.71	0.78	0.78

^a^Values on the diagonal are the square roots of average variance extracted and the off-diagonal values are the correlation coefficients between the construct variables.

^b^PE: performance expectancy.

^c^EE: effort expectancy.

^d^SI: social influence.

^e^FC: facilitating conditions.

^f^HC: health consciousness.

^g^TR: trust.

^h^INT: intention to use.

^i^not applicable.

### Structural Model

After the measurement model was satisfied, the structural model was evaluated, and it was well converged. The results investigated the chi-square of the structural model, ratio of chi-square to *df*, goodness-of-fit index, adjusted goodness-of-fit index, normed fit index, comparative fit index, root mean square residual, and root mean square error of approximation. Table 3 presents the model fit indicators with their respective criteria as follows: (1) the comparative fit index was 0.91 (greater than 0.90), (2) the root mean squared error of approximation was 0.03 (smaller than 0.08), and (3) the goodness-of-fit index was 0.93 (greater than 0.90) [25-29]. These indicators were acceptable and showed good fit of the model to the data.

**Table 3:** Fit statistics.

Fit measures	Sample value	Recommended value
χ^2^/*df *^a^	2.71	<5.0 [26]
Goodness-of-fit index	0.93	≥0.90 [27]
Adjusted goodness-of-fit index	0.94	≥0.90 [27]
Normed fit index	0.91	≥0.90 [25]
Comparative fit index	0.91	≥0.90 [28]
Root mean square error of approximation	0.03	<.08 [29]
Square multiple correlation intention	0.76	N/A^b^

^a^χ^2^/*df*: chi-square distribution is a special gamma distribution, which is one of the most widely used probability distributions in statistical inferences, such as hypothesis testing and CI calculations.

^b^N/A: not applicable.

### Hypothesis Testing

**Figure 2:**
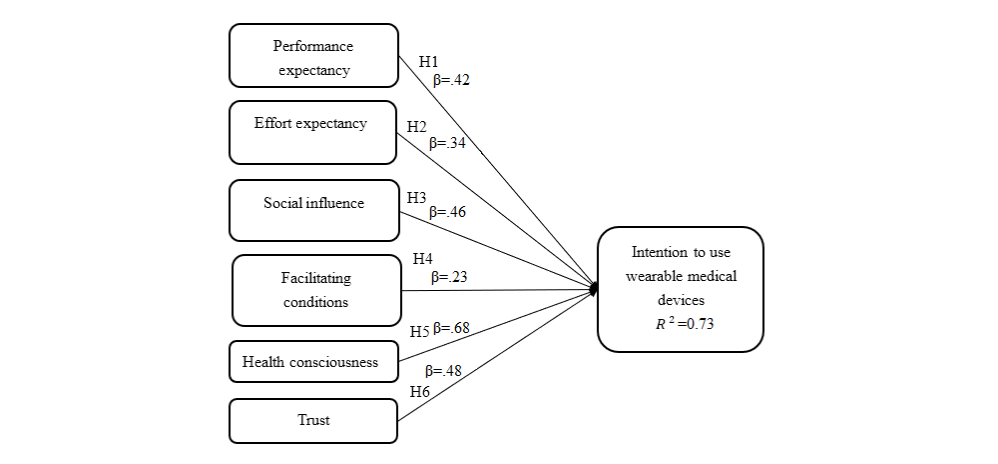
Results of the testing model. For all values, *P*<.001.

Significance was determined by running bootstrapping calculations with 352 samples and no sign variation. Four paths were relevant as shown in Figure 2.

Figure 2 shows the graphic description and the numerical results of the path coefficients. There were significant effects by PE (β=.42; *P*<.001), EE (β=.34; *P*<.001), SI (β=.46; *P*<.001), FC (β=.23; *P*<.001), health consciousness (β=.68; *P*<.001), and trust (β=.48; *P*<.001). The coefficients of these variables were statistically significant (*P*<.001) and had the expected signs (Figure 2). All hypotheses were supported.

## Results

According to the research findings, the various statistics confirmed that the revised UTAUT model was supported. The study provided some valuable insights into users’ intentions of wearable medical devices from their perspectives. PE, EE, SI, FC, health consciousness, and trust greatly influenced the intention to use wearable medical devices.

## Discussion

### Implications for Research

The results of this study provide several implications for researchers and practitioners. First, the results reveal the magnitudes of the impacts of the variables in the well-accepted revised UTAUT model in the context of the medical industry, particularly in the setting of wearable medical devices. Indeed, PE, EE, SI, and FC lead to positive intentions to use wearable medical devices, supporting H1, H2, H3, and H4.

Second, the impact of SI on adoption intention was more than that of FC, PE, EE, health consciousness, and trust, which is highly relevant in explaining the use of wearable medical devices. This implies that SI is an important factor affecting technology usage intention, that is, SI has positive effects on the intention of using wearable mobile devices. This finding differs from that of most studies on new health care technology acceptance and could reflect the culture, regulations, or rules in the Chinese social context. The result is also consistent with the findings of Ye et al [30]. Compared with another report by the author [31], PE has more positive effects on the intention to use library apps than UTAUT factors. Perhaps in different research backgrounds, the explanatory power of each factor of UTAUT would also be different.

Finally, this study modiﬁed the UTAUT by including constructs from health consciousness and trust. H5 and H6 were supported. The path coefficients were relevant, so the additional effects of health consciousness and trust were present. In particular, the degrees of health consciousness and trust were positive for strongly influencing the effects of the usage intention of wearable medical devices. In other words, this study demonstrated that higher health consciousness and trust can lead to much stronger intentions for using wearable medical devices. This study introduced health consciousness and trust as predictors in the Chinese social context to reflect the health care context, and this result is consisted with the findings in the studies by Dou et al [32] and Andrews et al [33].

### Implications for Practice

The implications of this study for practice are twofold. One practical implication is that based on the findings of the research model, service providers can make an effort to design a frequently well-used interface in order to enhance users’ PE, EE, and FC regarding the intentions of using wearable medical devices. In addition to the roles of PE, EE, and FC in usage intentions, SI has a positive effect on the intentions of using wearable medical devices. Service providers may still encourage users to spread positive word-of-mouth information (eg, positive ratings) to increase peer use. Another practical implication is that the research presented in this paper demonstrates the effects of health consciousness and trust in the use of wearable medical devices. Service providers can consider how to develop a very complicated device that takes into account an individual’ s ability and cognition in order to better match the wearable medical device user’s needs.

### Limitations and Future Research

Although the research findings contribute to the practice of marketing, the study is characterized by several limitations that may provide opportunities for future research. One limitation of this study is that as the sample was obtained by considering wearable devices or websites, the number of participants aged above 50 years was relatively low. Their behavior might differ somewhat from the population average, and this may have biased the results. Another limitation is that our study was limited to the customer base of one country. Further research is needed to examine differences in the effects of consumer characteristics across cultures.

## Appendix

Multimedia Appendix 1 Final questionnaire.

## Abbreviations

EE: effort expectancy
FC: facilitating conditions
IoT: internet of things
IS: information service
IT: information technology
PE: performance expectancy
SEM: structural equation model
SI: social influence
UTAUT: unified theory of acceptance and use of technology
