# Supplementary material for: Exploring the Usage Intentions of Wearable Medical Devices: A Demonstration Study
Source: Interact J Med Res. 2020 Sep 18;9(3):e19776. doi: 10.2196/19776 (PMC7532464; doi:10.2196/19776)
Supplement: Multimedia Appendix 1 [file ijmr_v9i3e19776_app1.docx]

Multimedia Appendix 1

Performance expectancy [4,33]

PE1 I find wearable medical devices useful in my daily life.

PE2 Using wearable medical devices increases my chances of achieving tasks that are important to me.

PE3 Using wearable medical devices helps me accomplish tasks more quickly.

PE4 Using wearable medical devices increases my productivity.

Effort expectancy [4, 33]

EE1 Learning how to use wearable medical devices is easy for me.

EE2 My interaction with wearable medical devices is clear and understandable.

EE3 I find wearable medical devices easy to use.

EE4 It is easy for me to become skillful at using wearable medical devices.

Social influence [4,33]

SI1 People who are important to me think that I should use wearable medical devices.

SI2 People who influence my behavior think that I should use wearable medical devices.

SI3 People whose opinions that I value prefer that I use wearable medical devices.

Facilitating conditions [4, 33]

FC1 I have the resources necessary to use wearable medical devices.

FC2 I have the knowledge necessary to use wearable medical devices.

FC3 The wearable medical device is compatible with other technologies I use.

FC4 I can get help from others when I have difficulties using wearable medical devices.

Health consciousness [33]

HC1 I am aware of and very concerned about my health.

HC2 I would make efforts to manage my health.

Trust [20]

TR1 I would trust that with big data and deep learning, wearable devices could deliver a reliable report after analyzing my health images.

TR2 I would trust that wearable medical devices are more accurate and reliable than human ophthalmologists, because they do not make subjective or empirical errors.

TR3 I would trust that stakeholders and reliable third parties would ensure the security and privacy of my personal data, health information, and images.

Usage intention [4,33]

BI1 I intend to use wearable medical devices in the future.

BI2 I will always try to use wearable medical devices in my daily life.

BI3 I plan to use wearable medical devices in future.

BI4 I predict I would use wearable medical devices in the future.
